# The Epidemiology and Clinical Spectrum of Melioidosis: 540 Cases from the 20 Year Darwin Prospective Study

**DOI:** 10.1371/journal.pntd.0000900

**Published:** 2010-11-30

**Authors:** Bart J. Currie, Linda Ward, Allen C. Cheng

**Affiliations:** 1 Tropical and Emerging Infectious Diseases Division, Menzies School of Health Research, Casuarina, Northern Territory, Australia; 2 Infectious Diseases Department, Northern Territory Clinical School, Royal Darwin Hospital, Darwin, Northern Territory, Australia; The George Washington University Medical Center, United States of America

## Abstract

**Background:**

Over 20 years, from October 1989, the Darwin prospective melioidosis study has documented 540 cases from tropical Australia, providing new insights into epidemiology and the clinical spectrum.

**Principal Findings:**

The principal presentation was pneumonia in 278 (51%), genitourinary infection in 76 (14%), skin infection in 68 (13%), bacteremia without evident focus in 59 (11%), septic arthritis/osteomyelitis in 20 (4%) and neurological melioidosis in 14 (3%). 298 (55%) were bacteremic and 116 (21%) developed septic shock (58 fatal). Internal organ abscesses and secondary foci in lungs and/or joints were common. Prostatic abscesses occurred in 76 (20% of 372 males). 96 (18%) had occupational exposure to *Burkholderia pseudomallei*. 118 (22%) had a specific recreational or occupational incident considered the likely infecting event. 436 (81%) presented during the monsoonal wet season. The higher proportion with pneumonia in December to February supports the hypothesis of infection by inhalation during severe weather events. Recurrent melioidosis occurred in 29, mostly attributed to poor adherence to therapy. Mortality decreased from 30% in the first 5 years to 9% in the last five years (p<0.001). Risk factors for melioidosis included diabetes (39%), hazardous alcohol use (39%), chronic lung disease (26%) and chronic renal disease (12%). There was no identifiable risk factor in 20%. Of the 77 fatal cases (14%), 75 had at least one risk factor; the other 2 were elderly. On multivariate analysis of risk factors, age, location and season, the only independent predictors of mortality were the presence of at least one risk factor (OR 9.4; 95% CI 2.3–39) and age ≥50 years (OR 2.0; 95% CI 1.2–2.3).

**Conclusions:**

Melioidosis should be seen as an opportunistic infection that is unlikely to kill a healthy person, provided infection is diagnosed early and resources are available to provide appropriate antibiotics and critical care.

## Author Summary

Melioidosis is an occupationally and recreationally acquired infection important in Southeast Asia and northern Australia. Recently cases have been reported from more diverse locations globally. The responsible bacterium, *Burkholderia pseudomallei*, is considered a potential biothreat agent. Risk factors predisposing to melioidosis are well recognised, most notably diabetes. The Darwin prospective melioidosis study has identified 540 cases of melioidosis over 20 years and analysis of the epidemiology and clinical findings provides important new insights into this disease. Risk factors identified in addition to diabetes, hazardous alcohol use and chronic renal disease include chronic lung disease, malignancies, rheumatic heart disease, cardiac failure and age ≥50 years. Half of patients presented with pneumonia and septic shock was common (21%). The decrease in mortality from 30% in the first 5 years of the study to 9% in the last five years is attributed to earlier diagnosis and improvements in intensive care management. Of the 77 fatal cases (14%), all had known risk factors for melioidosis. This supports the most important conclusion of the study, which is that melioidosis is very unlikely to kill a healthy person, provided the infection is diagnosed early and resources are available to provide appropriate antibiotics and critical care where required.

## Introduction

Melioidosis is the clinical disease following infection with the soil and water bacterium *Burkholderia pseudomallei* [1], [2]. It occurs in humans and a wide variety of animals and is thought to usually follow percutaneous inoculation. In addition, inhalation of aerosolized bacteria probably occurs during severe weather events such as tropical storms, aspiration is documented with near drowning and ingestion can occur, especially in grazing animals but also from mastitis-associated infected breast milk [3], [4]. Zoonotic transmission is described but is exceedingly uncommon, as are person-to-person transmission, nosocomial transmission and laboratory-acquired infection.

While melioidosis can present as a rapidly fatal septicemic illness and *B. pseudomallei* is now considered a potential biothreat agent, there remain major gaps in understanding the global distribution, epidemiology and pathogenesis of this infection. The known endemic distribution of *B. pseudomallei* is expanding well beyond the traditional melioidosis-endemic regions of Southeast Asia and northern Australia, with recent case reports of melioidosis from the Americas, Madagascar, Mauritius, India and elsewhere in south Asia, China and Taiwan [5], [6]. It remains unclear to what extent this reflects true expansion of endemicity rather than unmasking of the long-standing environmental presence of the bacterium.

Since October 1989 we have prospectively documented all cases of melioidosis in the tropical “Top End” of the Northern Territory of Australia. We described the presentations of the first 252 cases after 10 years of the Darwin prospective melioidosis study [7] and we now present the findings from 540 cases over 20 years.

## Materials and Methods

This study was approved by the Human Research Ethics Committee of the Northern Territory Department of Health and Families and the Menzies School of Health Research (HREC 02/38) and data were analysed anonymously. The Top End has a population of around 150,000 in an area of 516,945 km^2^, with almost 125,000 living in the Northern Territory capital city of Darwin (12°S). All patients with culture-confirmed melioidosis in the Top End from October 1st 1989 until September 30th 2009 were included. Investigation, treatment and follow-up were supervised in all cases in consultation with the Infectious Disease Department at Royal Darwin Hospital, the 350 bed referral hospital for the Top End. We followed all patients until death or after completion of therapy. Hazardous alcohol use was defined as greater than an average daily consumption of six standard drinks (60 g alcohol total) for males and four (40g alcohol total) for females. Chronic lung disease was defined as a documented diagnosis of chronic obstructive airways disease. Chronic renal disease was defined as a creatinine of >150 umol/L (N. R.<90 umol/L) before the melioidosis illness or after completion of therapy if not previously documented. Septic shock was defined as the presence of hypotension not responsive to fluid replacement together with hypoperfusion abnormalities manifest as end organ dysfunction [8].

Patient details were stored in a database and analysed using Stata version 10 (Stata Corporation, Texas). Chi-squared or Fisher exact tests were used to assess categorical variables; p<0.05 was considered significant and risk ratios and 95% confidence intervals were then calculated. To identify associations with a fatal outcome and with presentation with pneumonia and with bacteremia we conducted multivariable logistic regression analyses with stepwise backwards elimination of patient demographic and risk factor variables, with odds ratios and 95% confidence intervals calculated.

## Results

There were 540 cases and 77 deaths (14%) attributable to melioidosis over the 20 years. Ages ranged from 8 months to 91 years (median 49 years). There were 26 children ≤15 years old (5%) and two of these died, one with congenital heart disease and one with severe rheumatic heart disease. 372 patients (69%) were male and 281 Indigenous Australians (52%). 262 patients (49%) lived in the suburbs of Darwin, 65 (12%) on rural properties (“blocks”) outside Darwin city, 37 (7%) in the regional towns of Katherine and Nhulunbuy and 169 (31%) in remote Indigenous communities. Four infections were considered acquired in sub-tropical central Australia and three were acquired elsewhere in tropical northern Australia outside the Northern Territory.

Table 1 shows patient risk factors and outcomes by risk factor. There were only 2 patients with confirmed HIV infection, although a small number were not tested and 1 patient was seropositive for HTLV-I. Mortality was significantly higher in those with chronic respiratory disease (19% vs 13%; risk ratio = 1.5 (95% CI 1.1–2.4); p = 0.048). Although no other individual risk factor, including diabetes, was predictive of mortality, the absence of any risk factors was strongly predictive of survival; of the 106 (20%) with no identified risk factor for melioidosis, only two died (2%); both elderly, aged 75 and 82 years, respectively.

**Table 1:** Risk factors for 540 patients with melioidosis.

	Patients		Deaths	
Risk factor	n	% of total	n	% who died
Diabetes	213	39%	33	15%^4^
Hazardous alcohol use	211	39%	33	16%^4^
Chronic lung disease	140	26%	27	19%^5^
Chronic renal disease	65	12%	13	20%^4^
Rheumatic heart disease and/or congestive cardiac failure	39	7%	9	23%^4^
Malignancy	31	6%	8	26%^4^
Immunosuppressive therapy and other immunosuppression^1^	31	6%	6	20%^4^
Kava use	27	5%	4	15%^4^
Other^2^	6	1%	2	33%^4^
No risk factors	106	20%	2^3^	2%^6^

1 Mostly patients taking prednisolone therapy. Two with HIV infection and 1 with HTLV-I infection.

2 Three with hepatitis B-related chronic liver disease, 1 with hemochromatosis and chronic liver disease, 1 with other chronic liver disease and 1 with multiple strokes and prior aspiration pneumonia.

3 Both elderly; ages 75 and 82 years old but no other apparent risk factors for melioidosis.

4 Not significant in comparison to those without the risk factor.

5 Risk ratio = 1.5 (95% CI 1.1–2.4) in comparison to those without chronic lung disease; p = 0.048.

6 Risk ratio = 0.11 (95%CI 0.03–0.44) in comparison to presence of any risk factor; p<0.001.

407 patients (75%) were considered to have exposure to environmental *B. pseudomallei* through their recreational activities and 96 (18%) had direct exposure through occupational activities including gardening and outdoor maintenance, plumbing, building construction, plant machine operation and military exercises. Only 103 (19%) had no evident environmental or recreational exposure. In 118 (22%) cases there was a specific exposure scenario that was considered the likely infecting event. These included skin wounds sustained whilst working outdoors or gardening, or while playing sports such as soccer and rugby on muddy playing fields, or while fishing in fresh water rivers, hunting such as chasing feral pigs through tropical savannah swamps and motor vehicle accidents involving wet soil exposure. Several cases were in disabled people who rarely ventured outside their accommodation but were potentially exposed to aerosolised bacteria during storms. Regional clusters of cases occurred following severe weather events such as the Katherine river flood in January 1998, and Category 5 tropical cyclone Thelma, which hit the Tiwi Islands in December 1998 [9]. One cluster with nine cases of melioidosis and four deaths was attributed to confirmed *B. pseudomallei* contamination of the un-chlorinated water supply in a remote Aboriginal community [10].

Overall 436 (81%; 95%CI 77%–84%; p<0.005) presented during the wet season (November 1^st^–April 30^th^) and mortality was higher in cases presenting in January (23/102 (23%) died; p = 0.007) than in other months. Pneumonia was a significantly more common presentation in the peak monsoonal months of December to February (172/280; 61%) than in the other 9 months (106/260; 41%; p<0.001).

Of all presentations, 461 (85%) were considered acute (defined as symptoms present for less than 2 months) and from recent infection. 60 (11%) were chronic in nature (defined as symptoms present for over 2 months; 25 pneumonia, 23 skin ulcer(s), 12 others). These chronic infections were considered to be mostly acquired during the current or preceding wet season, with the delay until presentation explaining some of the cases diagnosed during the dry season. 17 of the 53 (32%) cases who presented during the mid dry season months of June 1^st^ to September 30th fulfilling the definition for chronic melioidosis. Patients with chronic melioidosis were less likely to be diabetic than those with acute melioidosis (20% vs 42%; p<0.001), with 42% having no identified risk factor in comparison to 17% of those with acute disease (p<0.001). Only 1 of 60 patients (2%) with chronic melioidosis died (p<0.001).

The remaining 19 (4%) patients were thought to have reactivation of disease from a latent focus of *B. pseudomallei* infection, based on long-standing prior radiological abnormalities and/or known long-standing positive melioidosis serology (13 pneumonia, 2 bacteremia no focus, 2 genitourinary infection, 1 each soft tissue infection and skin abscess). Those with presumptive reactivated melioidosis were more likely to have underlying chronic lung disease (47% compared with 25% for all others; p = 0.03) and rheumatic heart disease and/or congestive cardiac failure (32% compared with 6% for all others; p<0.001) and 5/19 of these died (26% compared with 14% for all others; p = 0.13).

The clinical presentations and outcomes are shown in Table 2. Overall 298 (55%) patients were bacteremic. Pneumonia was the commonest principal clinical presentation on admission (278 cases; 51%), followed by genitourinary infection (76 cases; 14%) and skin infection (68 cases; 13%). There were 20 (4%) patients presenting with septic arthritis and/or osteomyelitis and 14 (3%) with neurological melioidosis, of whom 10 presented with meningo-encephalitis, 2 with myelitis and 2 with cerebral abscesses. Bacteremia without an evident clinical focus was also a common presentation (59 cases; 11%), with severity of illness ranging from rapidly fatal septic shock to a clinically very mild febrile illness. When septic shock occurred it was usually present on or within 24 hours of admission. Of the 116 patients (21%) with septic shock, 58 (50%) died from acute fulminant melioidosis. In contrast, for those without septic shock on presentation, mortality was 4% overall (19/424); even in the 195 of those without septic shock who were bacteremic, only 13 (7%) died. Of the 106 patients with no identified risk factor, 23 (22%) were bacteremic and 6 (6%) had septic shock and the only deaths in this group were the 2 elderly patients as already noted.

**Table 2:** Clinical presentations and outcomes of 540 cases of melioidosis.

	Total		Bacteremic		Non-bacteremic	
	Number	Deaths (Mortality)	Number	Deaths (Mortality)	Number	Deaths (Mortality)
**Septic shock**	**116 (21%)**	**58 (50%)**	**103**	**48 (47%)**	**13**	**10 (77%)**
Pneumonia	88	43 (49%)	78	35 (45%)	10^1^	8 (80%)
No evident focus	13	8 (62%)	12	7 (58%)	1^2^	1 (100%)
Genitourinary	10	5 (50%)	9	4 (44%)	1^3^	1 (100%)
Osteomyelitis/septic arthritis	4	2 (50%)	4	2 (50%)	0	0 (0%)
Soft tissue abscess	1	0 (0%)	0	0	1	0 (0%)
**Non-septic shock**	**424 (79%)**	**19 (4%)**	**195**	**13 (7%)**	**229**	**6 (3%)**
Pneumonia	190	12 (6%)	89	9 (10%)	101	3 (3%)
Skin infection	68	0 (0%)	1	0 (0%)	67	0 (0%)
Genitourinary	66	2 (3%)	41	2 (5%)	25	0 (0%)
No evident focus	52	2 (4%)	47	2 (4%)	5	0 (0%)
Soft tissue abscess(es)	18	0 (0%)	4	0 (0%)	14	0 (0%)
Osteomyelitis/septic arthritis	16	0 (0%)	10	0 (0%)	6	0 (0%)
Neurological	14	3 (21%)	3	0 (0%)	11	3 (27%)
**Total**	**540**	**77 (14%)**	**298 (55%)**	**61 (20%)**	**242 (45%)**	**16 (7%)**

1 7 blood cultures not done, 3 blood cultures negative.

2 Culture +ve for *B. pseudomallei* only from rectal swab, although fatal septic shock.

3 blood culture not done.

Table 3 shows significant risk factors in the 278 melioidosis patients with a primary presentation of pneumonia. On univariate analysis age ≥50 years, diabetes, excessive alcohol consumption and rheumatic heart disease and/or congestive cardiac failure were each associated with a propensity for presentation with pneumonia in comparison to other presentations. However on multivariable analysis diabetes and age were not independent predictors of a presentation with pneumonia, while chronic lung disease, excessive alcohol consumption and rheumatic heart disease and/or congestive cardiac failure were.

**Table 3:** Risk factors in 278 patients presenting with melioidosis pneumonia.

	Patients		Primary Pneumonia		Univariate			Multivariate		
	n	%	n	%	p	OR	95% CI	p	OR	95% CI
**Age ≥50 y**										
No	277	51%	130	47%					ns	
Yes	263	49%	148	56%	0.030	1.3	(1.04–2.0)			
**Diabetes**										
No	327	61%	156	48%					ns	
Yes	213	39%	122	57%	0.030	1.5	(1.04–2.1)			
**Hazardous alcohol use**										
No	329	61%	154	47%						
Yes	211	39%	124	59%	0.007	1.6	(1.1–2.3)	0.041	1.5	(1.02–2.1)
**Chronic lung disease**										
No	400	74%	181	45%						
Yes	74	26%	97	69%	<0.001	1.5	(1.3–1.8)	<0.001	2.5	(1.6–3.9)
**RHD/CCF**										
No	501	93%	248	50%						
Yes	39	7%	30	77%	0.001	3.4	(1.6–7.2)	0.009	2.8	(1.3–6.2)
**Kava use**										
No	513	95%	272	53%						
Yes	27	5%	6	22%	0.002	0.3	(0.1–0.6)	0.004	0.2	(0.1–0.6)

RHD/CCF; rheumatic heart disease and/or congestive cardiac failure.

ns; not significant.

Table 4 lists internal organ abscesses and other foci of infection. Following findings early in the study of frequent internal collections, CT scanning of abdomen and pelvis has been routinely performed on all patients with melioidosis since around 1995. Prostatic abscesses were present in 76 males (20%), the majority of which required drainage [11]. In comparison to case series from Thailand, hepatic abscesses were uncommon and as with splenic and renal abscesses rarely required drainage. Three women had mastitis and three men had epididymo-orchitis. Lymphadenitis (sometimes suppurating), muscle abscesses, diffuse myositis and cellulitis were all seen but were uncommon. Four patients had para-intestinal masses which were considered possible primary infection following ingestion of *B. pseudomallei*, as was a presentation with a ruptured large gastric ulcer with subphrenic abscess and suppurative peritonitis. Mediastinal widening on chest X-ray and CT scan was seen, sometimes with clearly enlarged mediastinal lymph nodes and usually in association with pneumonia (12/17 cases). Four patients had suppurative pericarditis, three with contiguous pulmonary infection and one without evident pulmonary infection who developed acute pericardial tamponade requiring emergency thoracotomy and a pericardial window. Two had mycotic pseudo-aneurysms and one woman presented with a ruptured uterus from a massive uterine wall abscess.

**Table 4:** Internal organ abscesses and other foci of infection in 540 patients with melioidosis.

Site	n	%
Prostatic abscess(es)	76	20%^1^
Splenic abscess(es)	28	5%
Kidney abscess(es)	18	3%
Liver abscess(es)	15	3%
Adrenal abscess	3	<1%
Psoas abscess(es)	4	<1%
Other muscle abscess/myositis	8	1%
Cellulitis	3	<1%
Lymphadenitis	9	2%
Mediastinal mass	17	3%
Pericarditis	4	<1%
Para-intestinal mass	4	<1%
Mastitis	3	<1%
Epididymo-orchitis	3	<1%
Mycotic (pseudo)aneurysm	2	<1%

1 calculated for males only.

In addition to the initial principal clinical presentation, subsequent clinically-evident secondary foci were not uncommon and examples are shown in Table 5. Secondary pneumonia was especially common in those presenting with genitourinary infection, septic arthritis/osteomyelitis and bacteremia without an apparent clinical focus, but was unusual in those presenting with skin infection. Secondary foci were also less common in those presenting with pneumonia, although brain abscesses and septic arthritis requiring surgery occurred in this group. Of note, the pattern of secondary neurological melioidosis was different from the encephalomyelitis seen as a primary presentation. Of the eight patients with secondary neurological disease, all were blood culture positive (in comparison to 3/14 of those with primary neurological melioidosis; p = 0.001) and 5 had abscesses (4 intracranial, 1 spinal cord).

**Table 5:** Secondary clinical foci for major primary diagnostic groups.

Primary diagnosis	Total number	Number with secondary foci (% of total)	More common secondary foci^1^
Pneumonia	278	41 (15%)	17 prostatic abscesses, 10 splenic abscesses, 7 septic arthritis, 6 neurological, 6 liver abscesses, 5 renal abscesses
Genitourinary	76	26 (34%)	17 pneumonia, 3 splenic abscesses, 2 liver abscesses
Skin infection	68	6(9%)	3 pneumonia, 2 splenic abscesses, 2 liver abscesses
Bacteremia, no initial focus	59	25 (42%)	13 pneumonia, 6 splenic abscesses, 4 septic arthritis, 3 prostatic abscesses, 2 liver abscesses, 2 osteomyelitis, 2 neurological, 1 renal abscess
Osteomyelitis/septic arthritis	20	11 (55%)	6 pneumonia, 3 splenic abscesses
Soft tissue abscess(es)	19	9 (47%)	3 pneumonia, 2 septic arthritis, 2 osteomyelitis, 2 splenic abscesses
Neurological melioidosis	14	5 (36%)	3 pneumonia

1 more than 1 secondary focus found in some cases.

121 patients (22%) were admitted to the Royal Darwin Hospital Intensive Care Unit (ICU) and of these 40 (33%) died. In the ICU 97 were ventilated (41 died; 42%) (Table 6) and 60 received granulocyte colony-stimulating factor (G-CSF) (15 died; 25%). Three patients also received activated protein C therapy (1 died). Of the 77 deaths overall, 75 were during the initial hospital admission, with the time from admission to death in these ranging from 0 to 111 days (median 3 days). Two patients were dead on arrival at hospital, 8 died on the day of admission, and 7 died the day after admission.

**Table 6:** Changing outcomes over the 20 years.

	First 5 years	Second 5 years	Third 5 years	Fourth 5 years	Total over 20 years
Number	88	164	139	149	540
Died (%)^1^	26 (30%)	24 (15%)	14 (10%)	13 (9%)	77 (14%)
Blood culture +ve (% of total)	40 (45%)	77 (47%)	83 (60%)	98 (66%)	298 (55%)
Septic shock (% of total)	16 (18%)	28 (17%)	39 (28%)	33 (22%)	116 (21%)
Septic shock and died (% mortality)^1^	16 (100%)	21 (75%)	12 (31%)	9 (27%)	58
Ventilated (% of total)	8 (9%)	22 (13%)	34 (24%)	33 (22%)	97 (18%)
Ventilated and died (% mortality)^2^	8 (100%)	16 (73%)	8 (24%)	9 (27%)	41
Median age (years)	51y	49y	49y	49y	49y
Indigenous (%)	40 (45%)	90 (55%)	63 (45%)	87 (59%)	
Diabetic (%)	37 (42%)	56 (34%)	55 (40%)	65 (44%)	213 (39%)
No risk factors	15 (17%)	39 (24%)	26 (19%)	26 (17%)	106 (20%)

1 Significant trend for lower mortality over time; p<0.001 Chi-squared test of trend for proportion.

2 Significant trend for lower mortality over time; p = 0.002 Chi-squared test of trend for proportion.

Of the 465 patients surviving the initial infection, 30 (6%) re-presented with culture-confirmed recurrent melioidosis subsequent to completion of antibiotic therapy, with 2 deaths in this group. Of these 30, 25 were considered to have relapse of an unsuccessfully eradicated infection, usually resulting from poor adherence to antimicrobial therapy. In these cases, the time from initial admission to first relapse was 3.6–28 months (median 8 months), with one of these patients dying. Two of these patients had a second relapses (25 and 27 months after their first relapse); one of these patients died during the second relapse and the other patient had a third relapse 5 years after the second relapse. For those surviving the initial admission, diabetes was more common in those who relapsed (16/25 (64%) vs 166/440 (38%); p = 0.016), as was bacteremia on admission (18/25 (72%) vs 221/440 (50%); p = 0.034).

There were 5 patients with recurrent melioidosis where the *B. pseudomallei* isolates were different from the original isolate by pulsed-field gel electrophoresis or multilocus sequence typing (MLST). One patient with cystic fibrosis had three separate presentations with melioidosis at ages 10, 14 and 18 years. There was a good response to therapy each episode, with each *B. pseudomallei* isolate being a different sequence type (ST) and frequent sputum cultures between episodes being consistently culture negative for *B. pseudomallei*. He was considered to have been re-infected on three separate occasions. Three other patients with recurrent melioidosis but disparate isolates on typing were also considered likely to have new infections, occurring 14, 58 and 72 months after the initial infection, respectively. One further patient had disparate isolates on MLST from presentations 8 months apart and was thought to have relapse of a probable initial infection with multiple *B. pseudomallei* strains, with the osteomyelitis of the second presentation being evident clinically during the first presentation. Clinically apparent re-infection with *B. pseudomallei* is therefore thought to have occurred in only 4/465 (1%) patients surviving the initial admission, despite most survivors remaining in the melioidosis-endemic location, with many having persisting risk factors and continuing environmental exposure to *B. pseudomallei*.

Of the 463 patients who did not die from melioidosis, all except one have eventually cleared their infection with antibiotic therapy. This is a patient with moderately severe bronchiectasis who presented with a productive cough at age 61 years, with *B. pseudomallei* cultured from sputum. Her sputum has remained consistently *B. pseudomallei* culture positive for 8 years, despite multiple courses of intravenous and prolonged oral antibiotics and also a lobectomy of the most severely bronchiectatic lung lobe. She nevertheless remains generally well.

Table 6 shows decreasing mortality over the 20 years of the study that was not explained by either increasing recruitment of less sick patients or fewer risk factors in patients. To further assess associations with mortality we included the following categorical variables in the initial logistic regression model; age (≥50 years), indigenous ethnicity, each of the risk factors from Table 1, location, and presentation in December, January or February. No individual risk factor was a significant independent predictor of mortality (data not shown), although in a similar logistic regression model with bacteremia as the outcome, independent predictors of bacteremia were indigenous ethnicity, age ≥50 years, diabetes, hazardous alcohol use, chronic renal disease, malignancy and immunosuppression (Table 7). Therefore our final model for mortality from melioidosis incorporated presence or absence of any of the defined risk factors as a dichotomous variable. In this model the independent factors associated with mortality were age ≥50 years (OR 2.0; 95% CI 1.2–3.3) and presence of any risk factor (OR 9.4; 95% CI 2.3–39), but not indigenous ethnicity, geographical location or season (Table 8).

**Table 7:** Associations with bacteremia.

	Patients		Bacteremic		Univariate			Multivariate		
	*n*	%	*N*	%	*p*	OR	95% CI	*p*	OR	95% CI
**Indigenous**										
No	259	48%	112	44%						
Yes	281	52%	186	67%	<0.001	2.7	(1.9–3.8)	<0.001	3.2	(2.1–4.9)
**Age ≥50 y**										
No	277	51%	131	48%						
Yes	263	49%	167	64%	<0.001	1.9	(1.4–2.7)	<0.001	2.3	(1.5–3.6)
**Diabetes**										
No	327	61%	152	47%						
Yes	213	39%	146	70%	<0.001	2.6	(1.8–3.7)	<0.001	2.2	(1.4–3.2)
**Hazardous alcohol use**										
No	329	61%	166	51%						
Yes	211	39%	132	64%	0.004	1.7	(1.2–2.4)	0.014	1.6	(1.1–2.4)
**Chronic renal disease**										
No	475	88%	247	53%						
Yes	65	12%	51	78%	<0.001	3.2	(1.8–6.0)	0.019	2.2	(1.1–4.3)
**Malignancy**										
No	509	94%	274	55%						
Yes	31	6%	24	77%	0.013	2.8	(1.2–6.6)	0.003	4.1	(1.6–11)
**Immuno-suppression**										
No	509	94%	275	55%						
Yes	31	6%	23	75%	0.036	2.4	(1.1–5.3)	0.028	2.7	(1.1–6.5)

**Table 8:** Associations with mortality.

	Patients		Deaths		Univariate			Multivariate		
	*n*	%	*n*	% died	p	OR	95% CI	p	OR	95% CI
**Age ≥50 y**										
No	277	51%	26	9%						
Yes	263	49%	51	19%	0.001	2.3	(1.4–3.9)	0.009	2.0	(1.2–3.3)
**Indigenous**										
No	259	48%	36	14%	ns			ns		
Yes	281	52%	41	15%						
**Location**										
Urban Darwin	262	49%	43	16%						
Rural Darwin	65	12%	5	8%	ns			ns		
Remote	169	31%	22	13%						
Other	44	8%	7	16%						
**Season**										
Mar–Nov	260	48%	28	11%	0.025	1.8	(1.1–2.9)	ns		
Dec–Feb	280	52%	49	18%						
**Risk factors** 1										
None	106	20%	2	2%						
Any	434	80%	75	17%	<0.001	11	(2.6–45)	0.002	9.4	(2.3–39)

1 Risk factors; as listed in Table 1.

ns; not significant.

## Discussion

Serological surveys suggest that most infections with *B. pseudomallei* are asymptomatic, with over half of teenagers seropositive in the highly endemic region of northeast Thailand [12]. It was estimated that for children in northeast Thailand approximately 1 in 4600 antibody-producing exposures results in clinical infection [13]. The Darwin prospective melioidosis study provides strong support for the vast majority of melioidosis cases being from recent infection, with 81% of cases presenting during the monsoonal wet season, similar to a figure of 75% in Thailand [14]. Nevertheless latency of *B. pseudomallei* with subsequent reactivation is well recognised, being described as long as 62 years after infection in a returned World War II prisoner of war infected in southeast Asia [15]. It was estimated from serology studies that following the Vietnam War around 225,000 US service personnel may have been infected with *B. pseudomallei* [16]. This was called the “Vietnamese time bomb”, but the subsequent number of melioidosis cases following return to the USA has been comparatively small. Reactivation from a latent focus was considered to have occurred in only 19/540 (4%) cases in the Darwin study.

We previously estimated the average annual incidence rate of melioidosis in the Top End of the Northern Territory to be 19.6 cases per 100,000 population, with an estimated rate in diabetics of 260 cases per 100,000/year [17]. Yearly rates between 1990 and 2002 ranged from a low of 5.4/100,000 in 1993 to a high of 41.7/100,000 in 1998, a year with two severe tropical cyclones with intense rainfall and winds. This compares to northeast Thailand, with an average annual melioidosis incidence rate between 1997 and 2006 of 12.7/100,000 and with a highest rate of 21.3/100,000 in 2006 [18].

In the Darwin study 75% of melioidosis cases reported recreational activities that would result in exposure to environmental *B. pseudomallei* and 18% had clear occupational exposure. Both males (69%) and indigenous Australians (52%) were over-represented, most likely reflecting increased environmental exposure. There were 118 cases (22%) where history revealed a likely specific infecting event. *B. pseudomallei* is common in the urban environment of Darwin and most of the 49% of patients in the study who lived in the city of Darwin were infected in the city environs, including domestic gardens and yards. Mortality in the Darwin study was not linked to geographical location, being actually higher (although not statistically significantly so) in the urban population than in the rural and remote population (Table 8). In contrast, in northeast Thailand 81% of cases of melioidosis were in rural rice farmers and their children [14]. In Singapore melioidosis has occurred in construction workers, gardeners and military personnel, but in that tropical island city state, where over 80% of people live in high-rise apartments, the reasons for infection often remain unclear [19].

Earlier in the Darwin prospective melioidosis study we established that the incubation period for acute melioidosis following specific infecting events was 1–21 days (mean, 9 days) [20]. The incubation period, clinical presentations of melioidosis and outcomes are thought to be determined by a combination of bacterial load infecting the individual, putative *B. pseudomallei* strain differences in virulence, mode of infection and, most importantly host risk factors for disease [21]. For instance, less severe disease with symptoms present for over 2 months before presentation (chronic melioidosis) was significantly less common in diabetics and was more commonly seen in those without underlying risk factors.

The association between inhalation as a route of acquisition and increased severity of disease with higher mortality than percutaneous exposure is well recognised for anthrax, plague and tularaemia, but appears to have been under-appreciated in melioidosis. While the association between melioidosis and rainfall is well established [14] and there is epidemiological support for inhalation of aerosolised *B. pseudomallei* during severe weather events resulting in a pneumonic presentation with higher mortality [9], [19], [22], the overall contribution of inhalation of *B. pseudomallei* in comparison to percutaneous inoculation remains entirely unclear. Support for inhalation of *B. pseudomallei* from this study includes that 61% of admissions during the peak monsoonal months of December to February were with pneumonia, in comparison to only 41% in the other 9 months, plus the recognition that mediastinal lymphadenopathy is not uncommon.

Diabetes is the most important risk factor for melioidosis, followed by hazardous alcohol use, chronic lung disease and chronic renal disease [7], [14], [17], [19], [23], [24], [25]. Malignancy, immunosuppression and thalassemia are also recognised risk factors [24]. In the Darwin study 39% of patients were diabetic, with nearly all having adult onset type 2 diabetes. Rates of diabetes from other endemic locations were 57% in the largest series from Thailand [24], 48% in Singapore [19], 60% in Taiwan [26], 38% in bacteremic melioidosis patients in Malaysia [27] and 42% in north Queensland, Australia [25]. When considering the estimated prevalence of diabetes in the whole population, we previously calculated the risk of melioidosis in diabetics in the tropical Top End of the Northern Territory to be 21.2 (95% CI 17.1–26.3) times the risk in non-diabetics [17], which is similar to data from Thailand [14].

A lack of association of melioidosis with HIV infection [28], [29] supports a limited role for adaptive immunity in protection against acquisition of and mortality from melioidosis, despite evidence for a cell-mediated immune response to *B. pseudomallei* [30], [31]. We proposed that a unifying hypothesis for the predominance of diabetes, excessive alcohol consumption and chronic renal disease in melioidosis patients was the critical role of innate immunity and especially robust neutrophil function in controlling infection with *B. pseudomallei* [7]. The specific defects in neutrophil function in diabetes, alcohol excess and renal disease have been well described and were the basis for trialling therapy with granulocyte-colony stimulating factor (G-CSF) in melioidosis [32], [33]. The dysfunctional neutrophil hypothesis is supported by a study in a mouse model showing a critical role for neutrophils in resistance to melioidosis [34] and a recent study from Thailand showing that, in comparison to non-diabetics, otherwise healthy diabetics had neutrophils displaying impaired phagocytosis of *B. pseudomallei*, reduced migration in response to interleukin-8 and an inability to delay apoptosis [35]. The occurrence of melioidosis in chronic granulomatous disease also supports a key role for neutrophils [36].

Our clinical impression is that the risk for melioidosis in those with hazardous alcohol use may often be directly related to binge drinking rather than chronic liver disease, with high blood alcohol levels at the time of exposure to *B. pseudomallei* inhibiting protection against bacterial propagation and dissemination. This is consistent with earlier studies on neutrophil function in alcohol intoxication [37], [38]. An additional potential pathogenetic mechanism for more severe disease in those with hazardous alcohol intake is the induction by alcohol of bacterial genes encoding various potential virulence mechanisms, as recently shown in transcriptional profiling studies of *Acinetobacter baumannii* grown in the presence of alcohol [39]. In the Darwin study hazardous alcohol use but not diabetes was an independent predictor of presentation with melioidosis pneumonia (Table 3). In addition to neutrophil dysfunction, alcohol excess also adversely affects many other components of innate pulmonary host defences, from decreased ciliary beat frequency to impaired alveolar macrophage phagocytosis and inhibited cytokine responses [40]. Various aspects of adaptive pulmonary immunity are also affected by alcohol, involving both cellular and humoral responses.

We have noted two comorbidities previously unrecognized as potential risk factors for melioidosis. Chronic lung disease was an independent predictor of pneumonic melioidosis, which may reflect defective innate immunity such as impaired alveolar macrophage function [41]. Rheumatic heart disease and cardiac failure may predispose to melioidosis by similar mechanisms [42].

It is being increasingly recognised that patients with cystic fibrosis are at substantial risk of infection with *B. pseudomallei* if they live in or travel to endemic regions [43]. Chronic infection can occur, with acute flares of pneumonia and progressive deterioration of lung function, as also seen with *B. cepacia* infection in cystic fibrosis [44], [45], [46]. Patients with cystic fibrosis should consider avoiding travel to locations where melioidosis is common. One Darwin patient with cystic fibrosis has had three separate infections with different genotypes of *B. pseudomallei*. In addition, there is only 1/463 survivors in the Darwin study in whom clearance of *B. pseudomallei* has not been possible. This patient has severe bronchiectasis and has had persisting pulmonary infection for 8 years; such inability to eradicate *B. pseudomallei* from sputum has only been previously documented in cystic fibrosis [43].

Around half of melioidosis cases present with pneumonia, which can be part of a fatal septicaemia, a less severe unilateral infection indistinguishable from other community-acquired pneumonias or a chronic illness mimicking tuberculosis [2], [47]. In the Darwin study mortality was 49% in those with pneumonia who also had septic shock, in comparison to 6% in those with pneumonia without septic shock and 4% in those with chronic pneumonia. Early clinical descriptions and animal studies showed that melioidosis pneumonia can follow percutaneous infection [48], but the proportions of our pneumonia cases which were from percutaneous exposure, inhalation or aspiration are unknown. Nevertheless the finding of mediastinal widening on chest X-ray and CT scan in some melioidosis patients is analogous to inhalational anthrax.

Other presentations in the Darwin study range from skin lesions without systemic illness [49], to overwhelming sepsis with abscesses disseminated in multiple internal organs. Genitourinary [11], bone, joint and neurological infections [50], [51], [52] are all well recognised. One manifestation of melioidosis commonly seen in Thailand [53], but not seen over the 20 years of the Darwin study is children presenting with parotid abscesses. The reasons for this difference remain unclear.

The dramatic presentation of melioidosis brainstem encephalitis or myelitis has been noted to be more commonly seen in Australia than in Thailand [1], [3]. Recent mouse studies have suggested that such neurological presentations may result from direct entry of *B. pseudomallei* to the brain from the nasal mucosa via the olfactory nerve or similar pathways [54]. Genetic differences between *B. pseudomallei* strains may account for regional clinical variations. It was recently demonstrated that the global *B. pseudomallei* population probably evolved from an ancestral Australian population which subsequently spread to Southeast Asia [55]. One possible explanation for the neurological disease being more common in Australia is differences in propensity between *B. pseudomallei* populations for actin-based motility of bacteria along nerve pathways, conferred by variants of the BimA gene which have been found to be geographically restricted [56]. The concept of direct brain invasion by *B. pseudomallei* in the Darwin cases of primary melioidosis meningo-encephalomyelitis is supported by the low bacteremia rate in comparison to all 8 of those with secondary brain infections being bacteremic.

Most of the less common presentations of melioidosis seen in the Darwin study have also been described from other locations. These include mycotic aneurysms [57], epididymo-orchitis [58], pericarditis [59] and mastitis with maternal to child transmission of melioidosis [4]. The common presence of diverse internal organ abscesses necessitating routine imaging is also well recognised [1], [60], [61]. In contrast to Thai studies, where spleen and liver abscesses predominate [60], prostate abscesses were extremely common in the Darwin series, being present in 76/372 (20%) males. While liver, spleen and renal abscesses responded to prolonged antibiotic therapy, prostatic abscesses usually required drainage, whether primary or secondary [11]. Although unusual, the presence of para-intestinal masses supports that ingestion can occasionally be the primary route of infection in humans, as is more commonly seen in grazing animals [62].

Whatever the initial clinical presentation, secondary foci of infection are common in melioidosis (Table 5), presumably from bacteremic spread and reflecting the high rate of bacteremia overall (55%). Of those 59 patients presenting with bacteremia without an apparent focus, 25 (42%) subsequently developed an evident secondary focus of infection. Secondary pneumonia and septic arthritis were especially common.

Therapy of melioidosis requires prolonged antibiotics to cure infection and prevent relapse [63]. In the Darwin study 25/465 (5.4%) patients who survived the initial infection relapsed after treatment, with a median time to relapse of 8 months from initial admission, in comparison to 86/889 (9.7%) and 6 months from commencement of oral therapy in Thailand [24]. Choice and duration of and compliance with antibiotic therapy were the strongest indicators of risk for relapse in both locations. Diabetes was significantly associated with risk for relapse in the Darwin series but not in Thailand, while in both locations bacteremia on initial admission was associated with relapse, although only significantly so in Thailand.

Genotyping of *B. pseudomallei* from recurrent melioidosis has shown that reinfection can also occur but is less common than relapse [24], [64]. In northeast Thailand reinfection occurred in 30/899 (3.4%) patients, making the incidence of melioidosis reinfection substantially higher than that of primary infection [24]. This is in contrast to the Darwin patients, where reinfection was documented in only 4/465 (1%), despite most having persisting risk factors and continuing exposure and even raising the possibility of some acquired immunity to reinfection following melioidosis. Simultaneous infection with more than one strain of *B. pseudomallei* has been shown to very uncommon (2/133 cases in Thailand) [65], but was thought likely in one of the patients in this study.

Mortality from melioidosis in the Darwin study was 14%, with 75 of the 77 deaths occurring during the initial hospital admission and only 2 deaths from relapsed melioidosis. Mortality during the first 5 years of the study (from October 1989) was 30% and during the last 5 years (until October 2009) was 9% (p<0.001). These rates compare with 49% mortality in the large Thai study from 1986–2004 [24], with mortality now decreasing in that region [18], 65% in bacteremic patients in Malaysia during 1976–1991 [27], 16% in Singapore between 1998–2007 [19], 22% in Taiwan between 2000 and 2005 [26] and 25% in north Queensland between 1996 and 2004 [25].

The decreasing mortality over the 20 years of the Darwin study cannot be attributed to ascertainment bias from improved diagnosis of less severe cases. Indeed, the bacteremia rate was higher in the last 5 years (66%), possibly reflecting both more frequent repeat culturing in suspected cases and improved laboratory detection of low level bacteremia. The median age, rates of septic shock, percentages with various risk factors and the proportion with no risk factors did not change significantly over the 20 years (Table 6). The overall bacteremia rate of 55% compares with up to 65% in Thailand [24] [66], 50% in Singapore [19] and 60% in north Queensland [25].

We attribute the improved survival over time to a combination of earlier diagnosis of melioidosis through increased community and health staff awareness of the possibility, earlier treatment with ceftazidime or meropenem [67] and probably most importantly, access to and improvements in intensive care management of the septic patient. In many melioidosis-endemic regions renal replacement therapy and other resources for managing the metabolic abnormalities and organ dysfunction seen in severe sepsis are limited and without these mortality in septicemic melioidosis will remain high [2], [24], [68]. A greater proportion of patients were ventilated in the second half of the Darwin study and mortality in patients with septic shock was 100% in the first five years and decreased to 27% in the last 5 years (Table 6). Our initial optimism of potential benefit from G-CSF therapy in septicemic melioidosis [32] has been tempered by a randomized controlled trial in Thailand which showed that G-CSF conferred no mortality benefit in severe melioidosis in that setting [33]. Nevertheless those treated with G-CSF in that study had a longer duration of survival, suggesting that if state-of-the-art ICU therapy is available G-CSF may be beneficial.

A decrease in mortality in melioidosis similar to that seen in Darwin has also occurred in Singapore, where mortality in 1989–1996 was 40% in comparison to the more recent 16% [19]. It is notable that during the more recent series from Singapore there was a period in March–April 2004 with case numbers, proportion with pneumonia (83%) and mortality (53%) all higher than at other times [19]. This cluster of cases followed heavy rainfall and strong winds. Genotyping showed a diversity of strains, excluding a point source outbreak and the severe disease in this cluster was attributed to a possible shift to inhalation of aerosolized *B. pseudomallei* [19], [69]. This is analogous to clusters seen in the Darwin study following severe weather events [9].

This study provides strong support for the presence of specific host risk factors being the most important determinant of mortality from melioidosis. Older age is also recognised as a risk factor for melioidosis [14], [17], [19] and in the Darwin study age ≥50 years was an independent predictor of death from melioidosis (Table 8). Of the 77 deaths from melioidosis over the 20 years, 2 were in elderly patients without other evident risk factors and all of the other 75 fatal cases had at least one of the specific recognised risk factors listed in Table 1. That severe disease is very uncommon in melioidosis in patients without risk factors is evident from the much lower rates of bacteremia (22%) and septic shock (6%) in these patients in the Darwin study. The association of diabetes with bacteremia in patients with melioidosis has been noted in Thailand [23]. Nevertheless, while all the listed risk factors including diabetes were independently associated with bacteremia in the Darwin study (Table 7), no individual risk factor apart from age was an independent predictor of mortality. This reflects that 80% of melioidosis cases in the Darwin study had at least one risk factor irrespective of age and it was the presence of any of these risk factors that was highly predictive of mortality (OR 9.4; 95% CI 2.3–39).

Although the higher proportion of presentations with pneumonia during the peak monsoonal months of December to February supports a role for inhalation and although mortality was higher on univariate analysis during these 3 months, multivariate analysis showed that seasonality was not itself a significant independent predictor of mortality (Table 8). Furthermore, while severe melioidosis is associated with an array of pathogen induced immune dysregulation [70], that no death occurred in a patient without risk factors does not support an important role for cytokine-related human genetic polymorphisms in determining outcomes in melioidosis [71]. Therefore, although disease may be more severe following inhalation and/or higher infecting load of *B. pseudomallei*, the only predictor of mortality from melioidosis is the presence of defined risk factors such as diabetes, hazardous alcohol use, chronic lung or renal disease and older age.

In conclusion, melioidosis should be more seen as an opportunistic pathogen that is very unlikely to kill a healthy person, provided the infection is diagnosed early and resources are available to provide appropriate antibiotics and critical care where required.

## Supporting Information

Checklist S1 STROBE Checklist
(0.09 MB DOC)
