# Supplementary material for: The Epidemiology and Clinical Spectrum of Melioidosis: 540 Cases from the 20 Year Darwin Prospective Study
Source: PLoS Negl Trop Dis. 2010 Nov 30;4(11):e900. doi: 10.1371/journal.pntd.0000900 (PMC2994918; doi:10.1371/journal.pntd.0000900)
Supplement: Checklist S1 — STROBE Checklist (0.09 MB DOC) [file pntd.0000900.s001.doc]

STROBE Statement—Checklist of items that should be included in reports of ***cohort studies***

|  | Item No | Recommendation |
| --- | --- | --- |
| **Title and abstract** | 1 | (*a*) Indicate the study’s design with a commonly used term in the title or the abstract **YES PAGE 1** |
|  |  | (*b*) Provide in the abstract an informative and balanced summary of what was done and what was found **YES PAGES 2-3** |
| Introduction | | |
| Background/rationale | 2 | Explain the scientific background and rationale for the investigation being reported **YES – PAGE PAGE 5** |
| Objectives | 3 | State specific objectives, including any prespecified hypotheses **YES OBJECTIVE PAGE 5, NO HYPOTHESES HOWEVER BUT DISCUSSION IS EXTENSIVE IN DISCUSSING THE IMPORTANT (AND OFTEN NEW FINDINGS)** |
| Methods | | |
| Study design | 4 | Present key elements of study design early in the paper **YES – PAGES 6-7** |
| Setting | 5 | Describe the setting, locations, and relevant dates, including periods of recruitment, exposure, follow-up, and data collection **YES – AS ABOVE** |
| Participants | 6 | (*a*) Give the eligibility criteria, and the sources and methods of selection of participants. Describe methods of follow-up **YES – AS ABOVE. NOTE THAT FOLLOW UP REPORTED IN THIS PAPER IS PRINCIPALLY DEATH/SURVIVAL FROM MELIOIDOSIS. THE EMPHASIS IS OTHERWISE ON DEMOGRAPHY, PRESENTATION.** |
|  |  | (*b*) For matched studies, give matching criteria and number of exposed and unexposed **N/A** |
| Variables | 7 | Clearly define all outcomes, exposures, predictors, potential confounders, and effect modifiers. Give diagnostic criteria, if applicable **YES – SEE RESULTS, TABLES AND DISCUSSION** |
| Data sources/ measurement | 8* | For each variable of interest, give sources of data and details of methods of assessment (measurement). Describe comparability of assessment methods if there is more than one group **YES – SEE METHODS** |
| Bias | 9 | Describe any efforts to address potential sources of bias **ALL CASES OF CULTURE +VE MELIOIDOSIS WERE INCLUDED IN THE STUDY. DISCUSSION DOES COVER POTENTIAL BIASES THAT MAY HAVE IMPACTED ON THE IMPROVED SURVIVA; SEE SPECIFICALLY PAGE 23** |
| Study size | 10 | Explain how the study size was arrived at **YES** - **ALL CASES OF CULTURE +VE MELIOIDOSIS OVER THE 20 Y WERE INCLUDED IN THE STUDY** |
| Quantitative variables | 11 | Explain how quantitative variables were handled in the analyses. If applicable, describe which groupings were chosen and why **YES** - **SEE METHODS AND RESULTS AND DISCUSSION** |
| Statistical methods | 12 | (*a*) Describe all statistical methods, including those used to control for confounding **YES -SEE METHODS AND RESULTS RE THE MULTIVARIABLE ANALYSES** |
|  |  | (*b*) Describe any methods used to examine subgroups and interactions **THERE WERE NO** **SUBGROUPS WITHIN THE MAIN VARIABLES INCLUDED IN ANALYSIS** |
|  |  | (*c*) Explain how missing data were addressed **APART FROM THE PATIENTS WHO DID NOT HAVE BLOOD CULTURES TAKEN (SEE TABLE 2 FOR NUMBERS), THERE WERE NO MISSING DATA IN THE VARIABLES ANALYSED FOR THIS PAPER, INCLUDING THE MULTIVARIABLE ANALYSES – THAT IS PART OF THE STRENGTH OF THIS 20 YEAR PROSPECTIVE STUDY** |
|  |  | (*d*) If applicable, explain how loss to follow-up was addressed **N/A – ALL PATIENTS INCLUDED AND ACCOUNTED FOR** |
|  |  | (*e*) Describe any sensitivity analyses **N/A** |
| Results | | |
| Participants | 13* | (a) Report numbers of individuals at each stage of study—eg numbers potentially eligible, examined for eligibility, confirmed eligible, included in the study, completing follow-up, and analysed **AS ABOVE – ALL 540 PATIENTS INCLUDED** |
|  |  | (b) Give reasons for non-participation at each stage |
|  |  | (c) Consider use of a flow diagram **N/A** |
| Descriptive data | 14* | (a) Give characteristics of study participants (eg demographic, clinical, social) and information on exposures and potential confounders **YES – SEE RESULTS** |
|  |  | (b) Indicate number of participants with missing data for each variable of interest **AS ABOVE – N/A** |
|  |  | (c) Summarise follow-up time (eg, average and total amount) **YES; OUTCOME WAS DEATH FROM MELIOIDOSIS AND TIME TILL DEATH IS DESCRIBED IN RESULTS** |
| Outcome data | 15* | Report numbers of outcome events or summary measures over time **YES** |
| Main results | 16 | (*a*) Give unadjusted estimates and, if applicable, confounder-adjusted estimates and their precision (eg, 95% confidence interval). Make clear which confounders were adjusted for and why they were included **YES – SEE RESULTS AND TABLES** |
|  |  | (*b*) Report category boundaries when continuous variables were categorized **YES - SEE AGE BOUNDARIES IN RESULTS AND TABLES** |
|  |  | (*c*) If relevant, consider translating estimates of relative risk into absolute risk for a meaningful time period **NOT RELEVANT FOR THIS PAPER** |
| Other analyses | 17 | Report other analyses done—eg analyses of subgroups and interactions, and sensitivity analyses **AS ABOVE – N/A** |
| Discussion | | |
| Key results | 18 | Summarise key results with reference to study objectives **YES** |
| Limitations | 19 | Discuss limitations of the study, taking into account sources of potential bias or imprecision. **SEE 9 ABOVE RE MORTALITY** Discuss both direction and magnitude of any potential bias |
| Interpretation | 20 | Give a cautious overall interpretation of results considering objectives, limitations, multiplicity of analyses, results from similar studies, and other relevant evidence **YES - DONE** |
| Generalisability | 21 | Discuss the generalisability (external validity) of the study results **YES – THE MORTALITY DIFFERENCE BASED ON AVAILABLE HEALTH RESOURCES (EG ICU) IS DISCUSSED** |
| Other information | | |
| Funding | 22 | Give the source of funding and the role of the funders for the present study and, if applicable, for the original study on which the present article is based **YES** |

*Give information separately for exposed and unexposed groups.

**Note:** An Explanation and Elaboration article discusses each checklist item and gives methodological background and published examples of transparent reporting. The STROBE checklist is best used in conjunction with this article (freely available on the Web sites of PLoS Medicine at http://www.plosmedicine.org/, Annals of Internal Medicine at http://www.annals.org/, and Epidemiology at http://www.epidem.com/). Information on the STROBE Initiative is available at http://www.strobe-statement.org.
